# The Immunomodulatory Properties of Mesenchymal Stem Cells

**DOI:** 10.1155/2018/9214831

**Published:** 2018-12-20

**Authors:** Junji Xu, Dunfang Zhang, Peter Zanvit, Dandan Wang

**Affiliations:** ^1^Molecular Laboratory for Gene Therapy and Tooth Regeneration, Beijing Key Laboratory of Tooth Regeneration and Function Reconstruction, Capital Medical University School of Stomatology, Beijing, China; ^2^National Institute of Dental and Craniofacial Research, National Institutes of Health, Bethesda, Maryland, USA; ^3^Sichuan University, Chengdu, China; ^4^Department of Rheumatology and Immunology, The Affiliated Drum Tower Hospital of Nanjing University Medical School, Nanjing, China

Mesenchymal stem cells (MSCs) were initially described as a group of multipotent, self-renewal cells derived from bone marrow, adipose tissue, umbilical cord, dental pulps, and other tissues. Although there are different voices recently about the name or concept of MSCs, studies in the latest decades showed that these groups of cells could mediate a variety of immunomodulatory properties, which may affect both innate and adaptive immune responses. Moreover, dysfunction of the immunoregulatory function of MSCs has been suggested to play a role in the pathogenesis of autoimmune and inflammatory diseases. Therapeutic effects of MSCs on experimental and clinical autoimmune diseases and inflammation including Sjogren's syndrome, Crohn's disease, systemic lupus erythematosus, and rheumatoid arthritis have also been reported. However, the mechanisms mediating the immunosuppressive effects of MSCs remain incompletely understood.

Here, we highlight some of the critical ongoing challenges published in this special issue. Y. Su et al. found that the impairment of immunoregulatory function in MSCs from Sjogren's syndrome was because of the higher expression of BMP6. Their result showed that BMP6 could downregulate PGE2 secretion from MSCs via Id1 (inhibitor of DNA-binding protein 1); neutralizing BMP6 and knockdown of Id1 could restore the BMMSC immunosuppressive function both in vitro and in vivo. L. Guo et al. found that D-mannose could affect the immunomodulation of tooth MSCs. Their results showed that MSCs pretreated by D-mannose have stronger inhibition function on T cell proliferation and induce more T cells to differentiate into regulatory T cell by decreasing IL-6 production. F. Yan and O. Liu found that MSCs from the dental pulp can regulate natural killer cell function. Their results showed that MSCs could impair proliferation and promote apoptosis of activated NK cells and decrease cytotoxicity of activated NK cells via CD73 and adenosine. H. K. Lee et al. showed that prednisone or mycophenolate mofetil combined with MSCs has a better therapeutic effect than single therapy in lupus-prone MRL.*Fas*^lpr^ mice. Their result showed that this combination could synergistically inhibit T cell proliferation.

In this special issue, we present eight papers that address the issue about the multiple functions of postnatal MSCs, focusing on the investigation of immunomodulatory properties in these cells.

